# The Combined Effect of Biochar and Mineral Fertilizer on Triticale Yield, Soil Properties under Different Tillage Systems

**DOI:** 10.3390/plants11010111

**Published:** 2021-12-30

**Authors:** Luiza Usevičiūtė, Edita Baltrėnaitė-Gedienė, Dalia Feizienė

**Affiliations:** 1 Research Institute of Environmental Protection, Vilnius Gediminas Technical University, Saulėtekio al. 11, LT-10223 Vilnius, Lithuania; edita.baltrenaite-gediene@vilniustech.lt; 2 Lithuanian Research Centre for Agriculture and Forestry, Institute of Agriculture, Instituto al. 1, Akademija, LT-58344 Kėdainiai, Lithuania; dalia.feiziene@lammc.lt

**Keywords:** low-temperature biochar, Cambisols, mineral fertilizers, ploughless shallow tillage, direct drilling, pine wood, triticale

## Abstract

This study examined the effect of study time, biochar dose, and fertilization-tillage system on the improvement of sandy loam physical-chemical properties and triticale grain yield. The soil properties (water holding capacity (WHC), wettability, moisture content (MC), organic matter content (SOM), pH, and electrical conductivity (EC) were monitored in short time intervals (after 3, 6, 12, and 24 months). Soil was tilled in two methods (shallow ploughless tillage and direct drilling), fertilized with nitrogen, phosphorus, and potassium (NPK) fertilizers, and amended with three hydrophobic pine wood biochar doses (0 t/ha; 5 t/ha; 15 t/ha). It was found that 15 t/ha biochar dose had the highest effect on the soil’s physical-chemical properties improvement (SOM increased by 33.7%, pH—by 6.84%, EC—by 23.4%, WHC—by 8.48%, and MC—by 21.8%) compared to the variants without biochar. Direct drilling, fertilization with NPK fertilizers and 15 t/ha biochar dose significantly influenced the rise of soil’s physical-chemical properties and triticale yield (3.51 t/ha).

## 1. Introduction

The surface properties of soil have a high practical impact. They are closely interdependent. It is well known that many processes of interaction between soil particles with the outer environment occur through water which usually surrounds these particles in natural conditions. Interaction between solid material and liquid phases is one of the most important soil processes which include physical, chemical, and biological functions of soil [1]. Wettability is one of the most often occurring phenomena which arise between the surface boundaries of different phases [2]. It is a fundamental property controlling the wetting of plane and granular solid materials. Compared with the plane surfaces of the solid, wettability of granular materials has additional complexity of different level roughness effects (particle level or particle agglomeration level). The wettability of soil affects hydrological functions of soil systems including infiltration, preferential flow, and runoff. Control of surface wettability is equally important in various industrial applications [3]. Life and agricultural sciences pay much attention to the soil infiltration problem when the occurrence of hydrophobicity of soil decreases or temporarily weakens infiltration, which enhances runoff, erosion, or sedimentation of the surface.

It is known that soil wettability depends on its number of mineral/organic compounds and composition, fractions of different structures (sand, clay particles). Previous studies [2] found that the mineral part of the soil is characterized by the hydrophilic surface properties and organic matter which is described by the amphiphilic compounds and nonpolar organic components of the surface adsorbed on the surface of the particles and thus governing hydrophobicity of the soil solid phase. Free lipids including fatty acids, alcohols, alkanes, and suberin which are excreted from the plant roots all together contribute to the hydrophobicity of soil [4]. Some organic fractions which are related to the hydrophobicity of soil are humic acids, aliphatic fractions, and plant debris [5]. Minerals forming soil are considered wettable, since the free surface energy of inorganic soil particles is generally higher than water’s, and their contact angle values are usually close to 0°. In nature, the hydrophobicity of soil develops due to the formation of hydrophobic organic compounds or soil drying. On the other hand, a study driven by Vogelmann et al. [6] found that soil having the least amount of organic carbon had the highest severity of hydrophobicity; therefore, it can be stated that hydrophobicity is more related to the origin of these compounds but not with the amount. Hydrophobicity of soil is most usually considered as a natural phenomenon that occurs on the surface of sandy soils affected by wildfires or on the upper soil layers affected by some fungi species [7]. A study driven by Gonzalez-Penaloza [8] confirmed that higher hydrophobicity of soil is related to the course (sand) soil particles. Coarse structure soils have a lower surface area (in sandy soils from 0.01 to 0.1 m^2^/g) than fine structure soils (in clay soils from 5 to 750 m^2^/g), and some amount of hydrophobic organic compounds can induce higher hydrophobicity of coarse structure soil compared to fine structure soil due to its low specific surface area. Meanwhile, clay particles support the reduction in soil hydrophobicity by covering hydrophobic surfaces. This phenomenon (hydrophobicity of soil) can be found in different granular composition soils from many locations and climatic conditions, from sandy to clay soils, from slightly to severely eroded soils, from very acidic to strongly alkaline soils, from fertile to infertile soils [9]. 

The hydrophobicity of soil is related to other factors, such as the content of soil moisture as well pH [7]. Since hydrophobic compounds causing its hydrophobicity are of organic origin, some studies showed that there was no relation between the severity of the soil hydrophobicity and the amount of soil organic matter (SOM) [10]. However, Vogelmann et al. [6] found this link whilst with low coefficients of determination which prevent from making satisfactory forecasts of the possible occurrence of this phenomenon according to the amount of SOM. Diehl [11] determined that hydrolysis–condensation reactions of carboxylic and phenolic groups of SOMs can increase their wettability. Even though it is partly agreed of the origin of SOM compounds causing the hydrophobicity of soil, a precise mechanism of this phenomenon is still uncertain [12]. 

The wettability of soil can alter with time and it can affect soil geomorphological and hydrological processes. According to Doerr et al. [13], this variation depends on the amount of soil moisture and is related to wetting/drying cycles which originate due to seasonal variation of soil moisture. It is also important to note that the phenomenon of soil hydrophobicity does not occur constantly, since its maximal intensity is reached during the driest seasons and it can decrease or totally vanish during wet seasons [14]. Hubbert et al. [15] found the variation of soil hydrophobicity from average to severe hydrophobic when the soil was wet in the wintertime and when it had low moisture content during the summer season. A prolonged soil drying period increases the hydrophobicity level and, therefore, requires long soil re-wetting periods due to the restoration of soil wetting properties. Some studies showed that soil moisture had a strong negative correlation with the severity of hydrophobicity [16].

Previous studies found that variation of soil surface properties is highly impacted by the soil management practices, application of biochar, and fertilization with mineral fertilizers. Intensive soil tillage reduces the content of organic matter, induces the hydrophobicity of soil’s solid phase, and decreases the amount of water-stable aggregates—they govern the degradation of the structure of soil and impact the water flow processes through the soil profile [2]. Excessive decomposition of pellets can decrease soil porosity and aeration. Meanwhile, excessive usage of chemical fertilizers can induce many problems, such as the loss of nutrients, surface runoff, acidification or alkalinization of the soil, decrease of beneficial microbial populations [17]. These problems can be diminished after the application of biochar through the avoidance of soil compaction. Another advantage of biochar is that it can reduce the demand for fertilizers/excrements/compost and improve the retention of moisture, which decreases the need for frequent irrigation. Conservative tillage practices typically result in higher soil organic matter, reduced erosion, increased infiltration, and increased water-stable aggregates compared to traditional tillage practices. The impact of biochar on the soil quality in different management systems is not well understood yet. 

Triticale is a hybrid of wheat and rye that has been harvested due to a combination of positive wheat properties (yield efficiency, grain quality) and rye properties (disease resistance and durability) [18]. Triticale has been found to grow better than wheat in poor-quality soils. To date, triticale is mainly grown as fodder, cover crop, and for biogas production. Triticale is considered an interesting species that can be useful for cultivation even in unfavorable biotic and abiotic conditions [19]. Compared to wheat, triticale adapts better to a variety of soil and environmental conditions and can provide higher grain yields. Several factors influence the yield of triticale grains: local climatic and soil properties, uptake of mineral components, and appropriate methods of plant protection against diseases, pests, and weeds.

At present, there is not much information on the factors influencing triticale yield. In order to improve tillage practices, it is necessary to identify soil properties that control the variation of triticale grain yield. This study was conducted to determine the variation of selected soil physical and chemical properties from the field study and to evaluate the soil property that could explain the triticale grain yield. The objective of this study was to determine the effect of hydrophobic pinewood biochar dose and tillage-fertilization system on the improvement of sandy loam physical-chemical properties and triticale yield. Hypothesis—direct drilling, fertilization with NPK fertilizers, and 15 t/ha biochar dose has the highest effect on the improvement of sandy loam physical-chemical properties and triticale yield. Detailed and accurate information about the soil allows more precise control of soil properties and more cost-effective distribution of soil amendments.

## 2. Materials and Methods

### 2.1. Plot Description, Scheme

The experiment was conducted in the Institute of Agriculture at Lithuanian Research Centre for Agriculture and Forestry and in the Research Institute of Environmental Protection at Vilnius Gediminas Technical University (Vilnius Tech). The investigation was conducted in 2019–2021 (55°23′ N and 23°51′ E) in the long-term (20 years) soil tillage-fertilization systems field experiment. The soil was sandy loam (*Endocalcari-Epihypogleyic Cambisol* (FAO, 1998)). According to soil texture, this soil had the highest amount (53.7%) of sand (2–0.05 mm) particles, average amount (32.6%) of silt (0.05–0.002 mm) particles, and the lowest (13.7%) amount (<0.002 mm) of clay particles.

Research scheme: 

Factor A—soil tillage-fertilization system:

- S-1—ploughless shallow tillage (stubble cultivation at 10–12 cm + pre-sowing cultivation at 5–6 cm) and no fertilization with NPK fertilizers;
- S-2—ploughless shallow tillage and fertilization with NPK fertilizers;
- M-1—direct drilling (soil not tilled, direct drilling with disc drill having a rotary tiller) and no fertilization with NPK fertilizers;
- M-2—direct drilling and fertilization with NPK fertilizers.

Factor B—biochar dose:

- 0 t/ha;
- 5 t/ha;
- 15 t/ha.

Factor C—date of the investigation:

- 3 months;
- 6 months;
- 12 months;
- 24 months.

Biochar was incorporated into the soil before the sowing of summer triticale (*Triticum x Secale*) (on the 16 April 2019) during direct drilling with disc drill having a rotary tiller. The target yield of the summer triticale was 5 t/ha. Pine wood biochar (450 °C, 2 h) was used in the field experiment. Mineral fertilizers were applied: ammonium nitrate (34.5%), granular superphosphate (19%), and potassium chloride (60%). The experiment was arranged in a randomized complete block design with three replications (details of the experiment). 

**Figure 1:**
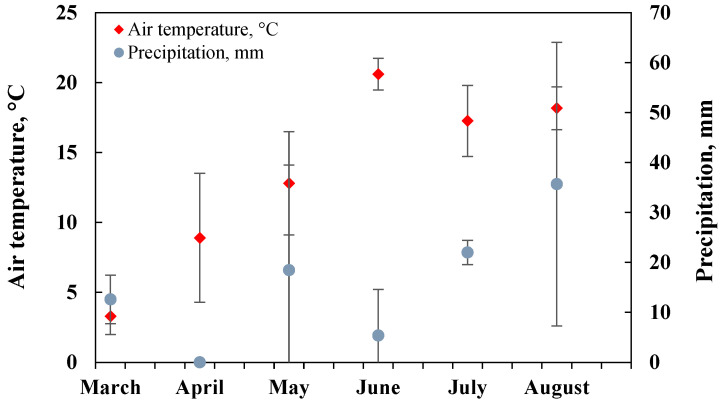
Average monthly temperature (°C) and amount of precipitation (mm) according to every month 10-day data from Dotnuva meteorological station, *n* = 3.

According to the meteorological data, March was the coldest month in the studied period of time (March-August) on the average (3.3 °C; long-term average –0.6 °C), and June was the warmest month (20.6 °C; long-term average 15.7 °C). There was no precipitation in April (long-term average is 37.6 mm), and the highest amount of precipitation was in August (35.67 mm; long-term average 73.2 mm) (Figure 1). In the summer season, June was the driest month (20.6 °C and 5.37 mm); therefore, during the experiment, soil became completely dry, and at the end of summer (in August) the highest amount of precipitation was observed compared to the overall studied period. 

### 2.2. Soil Sampling and Methodology for Hydro-Physical and Chemical Properties Determination

Soil samples were collected in four periods: 3, 6, 12, and 24 months after biochar application. Soil chemical and hydro-physical analyses were performed in the Research Institute of Environmental Protection. Soil samples were taken using a soil auger from every treatment from 0–15 cm soil layer. Plant residues were removed from samples before the analysis. Soil samples were dried in ambient conditions at 20 °C temperature and sieved through a 2 mm diameter sieve. The particle size distribution in soil samples was determined by using the volumetric particle size distribution method [20]. The wettability of soil was assessed by using the water drop penetration time test which is required for the complete drop infiltration [21]. The water holding capacity (WHC) of soil was determined and calculated according to the Formula (1) [22].

(1) $$\mathrm{WHC}=\frac{{\mathrm{mass}}_{\mathrm{saturated}}-{\mathrm{mass}}_{\mathrm{dry}}}{{\mathrm{mass}}_{\mathrm{dry}}}\times 100\%$$

The soil moisture content (MC) was calculated according to Formula (2) [22]:

(2) $$\mathrm{MC}=\frac{{\mathrm{mass}}_{\mathrm{wet}}-{\mathrm{mass}}_{\mathrm{dry}}}{{\mathrm{mass}}_{\mathrm{dry}}}\times 100\%$$

The soil pH was determined in soil: water suspension at ratio 1:1 using a pH meter [23]. The soil electrical conductivity (µs/cm) was also determined in soil: water suspension at ratio 1:1 according to the volume and using an electrical conductivity meter. The results of electrical conductivity represent the concentration of salt in the water of soil pores. The soil organic matter (SOM) was determined using the soil combustion method which is justified by dry (at 105 °C) soil combustion (at 550 °C), when constant sample weight is gained. The amount of SOM was calculated according to the mass difference before and after the combustion [24]. 

### 2.3. Biochar Production and Methodology for Determination of Its Physical-Chemical Properties 

Biochar was produced from pine wood biomass at 450 °C temperature and 2 h holding time in a muffle furnace (SNOL2000/2002, SnolTherm, Utena, Lithuania). For the analysis of the water drop penetration time test, 10 small droplets (0.04 mL) were laid down on the plane and dry biochar surface using a laboratory pipette, and time for the complete water drop penetration time (WDPT) was assessed. Biochar wettability can be classified as: hydrophilic (WDPT < 5 s), slightly hydrophobic (WDPT 5–60 s), strongly hydrophobic (WDPT 60–600 s), severely hydrophobic (WDPT 600–3600 s) and extremely hydrophobic (WDPT > 3600 s) [21]. For the determination of biochar pH, samples were mixed with 0.1 N KCl solution at a ratio of 1:10 [25]. After 10 min of shaking, the pH in biochar suspension was determined using a pH meter. For biochar electrical conductivity analysis, 20 g of the sample was placed in 200 mL of desalinated water and shaken for 1 h and then the solution was filtrated. Electrical conductivity was assessed in filtered water using a conductometer [26]. The biochar cation exchange capacity was analyzed using the ammonium acetate exchange method. Biochar elemental composition (C, H, N, O, S) was determined using a EuroEA3000-Single analyzer (EuroVector, Milan, Italy) [27]. The sample (dried and milled of 0.5–3 mg) was weighted directly into the small capsule which then was placed into the elemental analyzer. Concentration of potentially toxic elements (Pb, Zn, Cu, Cr, Cd and Ni) in biochar was analyzed using an atomic absorption spectrometer (AAS) (Buck Scientific, Norwalk, CA, USA). Dry biochar samples were combusted at 450 °C for 2.5 h until ashes. Then, every sample of 0.5 g was weighted and mixed with 3 mL of 65% of nitric acid and 9 mL of 37% of hydrochloric acid. After that, the solution was placed into the Milestone ETHOS acid digestion system (Milestone Srl, Milan, Italy). After the process, the obtained solution was placed into the flask, filtered, and diluted with deionized water until 50 mL [27]. After the filtration, the concentration of potentially toxic elements was determined using the AAS [28]. The biochar surface functional groups were determined using Fourier-transform infrared spectroscopy (FTIR) when wavelengths from 4000 to 450 cm^−1^ were used [29]. The biochar specific surface area was analyzed according to N_2_-Brunauer-Emmett-Teller (BET) theory and BET analyzer (Quantachrome Instruments, Boynton Beach, FL, USA) [30].

### 2.4. Comparative Characteristics of Pine Wood Biochar Physical-Chemical Properties 

**Figure 2:**
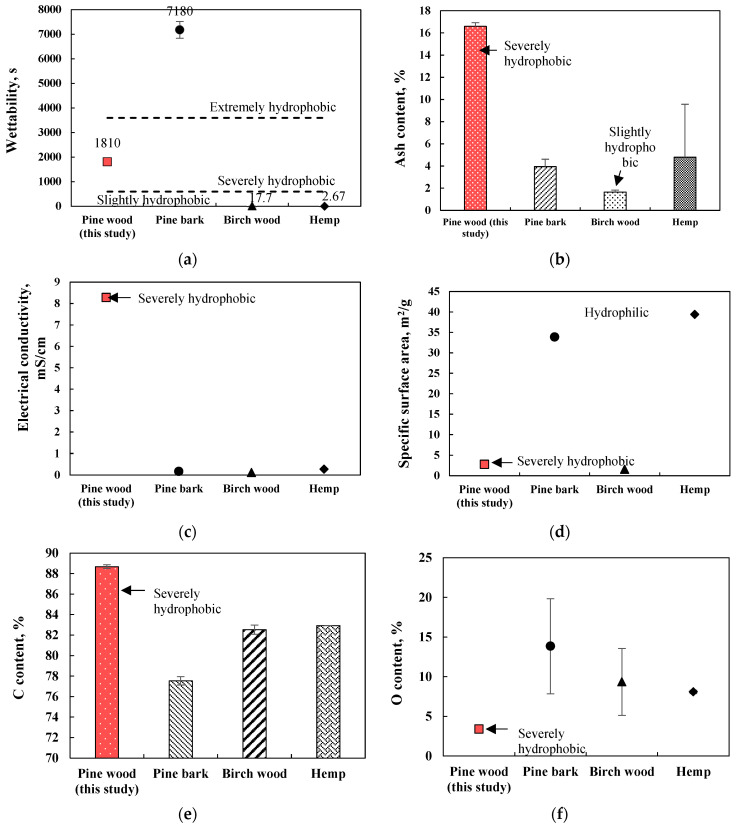
The comparison of physical-chemical properties of different biochar types, made from pine wood, birch wood, pine bark and hemp (450 °C, 2 h): (**a**) wettability (s), (**b**) ash content (%), (**c**) electrical conductivity (mS/cm), (**d**) specific surface area (m^2^/g), (**e**) C content (%), (**f**) O content (%), *n* = 3.

Pine wood low-temperature (450 °C) origin biochar had a high hydrophobicity feature (WDPT = 1810 s) which was higher compared to birch wood and hemp biochar, but lower than pine bark biochar (Figure 2a). Hydrophobic biochar in this study had a low specific surface area (2.77 m^2^/g; Figure 2d), low O content (3.39%; Figure 2f), high ash content (16.6%; Figure 2b), high electrical conductivity (8.28 µs/cm; Figure 2c), higher C concentration (88.7%; Figure 2e) and slightly higher pH (8.53; Table 1) compared to slightly hydrophobic birch wood and hemp biochar types and extremely hydrophobic pine bark biochar. The high hydrophobicity of pine wood biochar can be related to higher ash content which blocks pore space and inhibits water entry through the biochar surface. 

**Figure 3:**
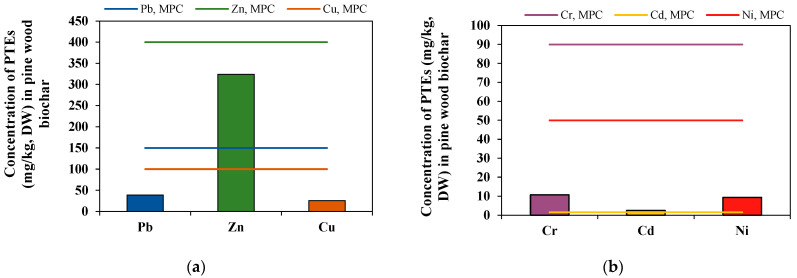
Concentrations of potentially toxic elements (mg/kg) in pine wood biochar: (**a**) lead (Pb), zinc (Zn) and copper (Cu), (**b**) chromium (Cr), cadmium (Cd) and nickel (Ni), *n* = 3.

Biochar types with higher initial ash content are less suitable for the soil amendment due to high amounts of potentially toxic elements (PTEs) which can cause soil pollution [31]. According to concentrations of potentially toxic elements, analyzed pine wood biochar corresponded to standard biochar quality considered in the European Biochar Certificate (EBC) in the case of five PTEs: Pb concentration was by 3.83 times lower compared to the standard biochar quality according to the maximum permissible Pb concentration (MPC, 150 mg/kg), Zn was 1.23 times lower, Cr was 8.39 lower, Cu was 3.89 times lower and Ni was 5.36 times lower (Figure 3a,b). According to cadmium concentration (2.48 mg/kg), the analyzed biochar slightly exceeded standard biochar quality limits according to the MPC (1.5 mg/kg). Some researchers claimed that Zn, Cu, and Pb are stabilized into the biochar [31]. For the long-term biochar application into the soil, it has to be carefully analyzed due to potentially toxic elements (PTEs) which can accumulate into the soil. According to the European Biochar Certificate, biochar cannot exceed the limit values of PTEs having an intention for its incorporation into the soil [32]. 

**Figure 4:**
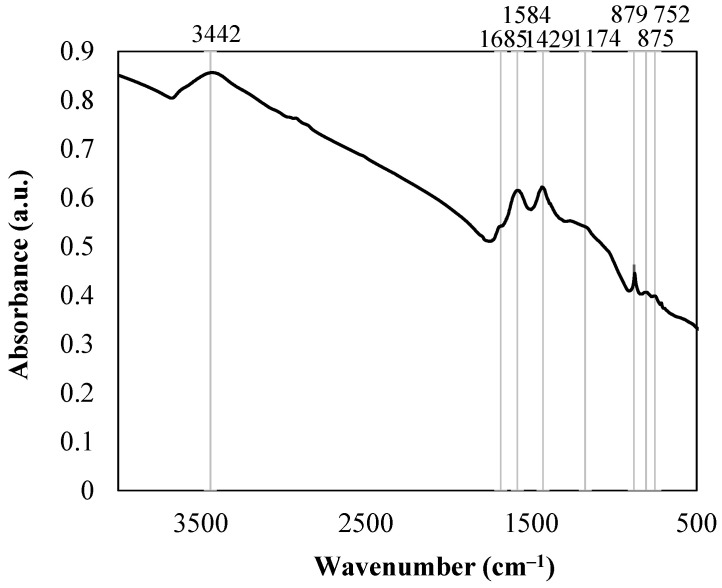
FTIR spectrum of low-temperature pine wood biochar, *n* = 1.

Biochar had eight peaks which shows the existence of some functional groups in the biochar structure: alcoholic –OH (3442 cm^−1^), acidic C=O (1684 cm^−1^), aromatic C=C (1684 cm^−1^, 1584 cm^−1^, 1429 cm^−1^), anhydride C–O (1174 cm^−1^) and aromatic C–H (805 cm^−1^, 879 cm^−1^, 752 cm^−1^) (Figure 4). FTIR spectrum of initial biochar showed strongly condensed biochar structure which can be seen from intensive C=C ring region [33]. It shows the growth of biochar aromaticity during the pyrolysis process. A rise in the peak at 879 cm^−1^ wavenumbers is related to C–H group deformations.

### 2.5. Statistical Analysis

Descriptive statistics (average value, standard deviation, maximum value, minimum value) was assessed using Microsoft Excel 2016 software. For the determination of a significant difference between treatments (different combinations of three factors: research date, tillage-fertilization system, and biochar rate) hydro-physical and chemical properties the three-factorial ANOVA was performed (package STATISTICA Base (version 6). Differences among studied groups were significant at *p* < 0.05 and *p* < 0.01. Additionally, the least significant difference (LSD_05_) was presented [29]. Correlation between soil hydro-physical and chemical properties and wettability which was expressed as intensity of absorption in hydrophilic functional groups was performed using Pearson correlation analysis [34]. Pearson’s correlation analysis of hydro-physical and chemical properties (soil organic matter, pH, electrical conductivity, water holding capacity, and moisture content) and C–O functional group intensity (a.u.) of soil were conducted using the SPSS software package (SPSS Inc., Chicago, IL, USA). 

## 3. Results

### 3.1. Soil Organic Matter Content

**Figure 5:**
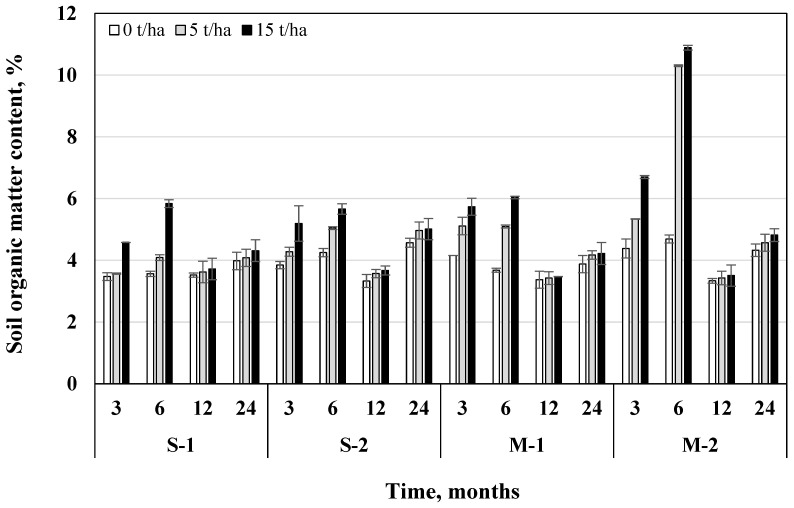
Influence of biochar on the amount of organic matter in different tillage-fertilization systems (S—ploughless shallow tillage, M—direct drilling, 1—unfertilized, 2—fertilized) after 3, 6, 12, and 24 months from the beginning of the experiment, *n* = 2.

Assessing the effect of time on soil organic matter (SOM) content, average data showed that after 24 months it was 6.14% lower than after 3 months, but 26% higher than after 12 months. Fertilization with nitrogen, phosphorus, and potassium (NPK) fertilizers resulted in higher SOM, which was 10.4% and 26.6% higher to unfertilized soil groups (in S and M tillage systems, respectively). On average, the application of 5 t/ha biochar dose increased SOM by 19.7%, and 15 t/ha by 33.7%, compared to the control groups (without biochar) (Figure 5).

The influence of biochar dose on SOM was significant in all tillage-fertilization systems (*p* < 0.05) (Table 2). Biochar usage in a direct drilling system (M) was more promising and had a greater effect on SOM compared to shallow ploughless tillage (S). If in the S system 5 t/ha biochar dose increased SOM by 5.63–11.7%, and the rate of 15 t/ha—by 26.9–22.2% (in S-1 and S-2 systems, respectively), then in the M system—18–41.3% and 29–54.8% (in M-1 and M-2 systems, respectively) compared to variants without biochar. 15 t/ha biochar dose determined the best SOM conditions only when it was used in combination with mineral fertilizers in both tillage systems (S—4.89% and M—6.48%, respectively). Regardless of the tillage-fertilization system, the biochar effect on SOM was most promising after 6 months, since SOM content was 51.6–75.5% higher in all variants compared to variants without biochar addition. After 3, 6, 12, and 24 months, variant with direct drilling + fertilization + 15 t/ha biochar dose (M-2) had the highest SOM (6.69%; 10.9%; 3.72%; 5.01%, respectively).

### 3.2. Soil pH

**Figure 6:**
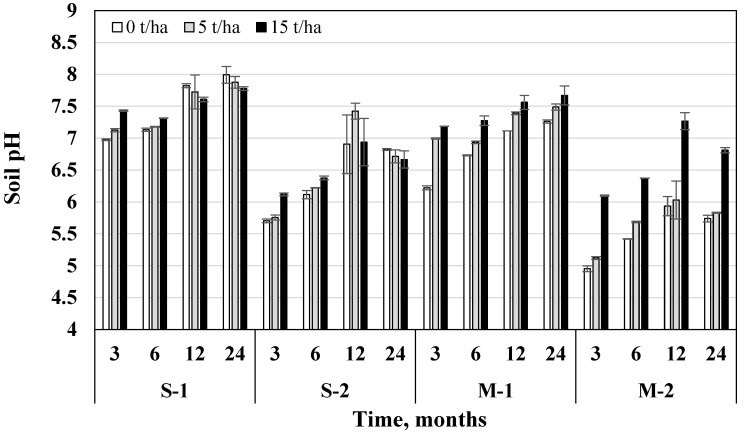
Influence of biochar on soil pH changes in different tillage-fertilization systems (S—ploughless shallow tillage, M—direct drilling, 1—unfertilized, 2—fertilized) after 3, 6, 12, and 24 months from the beginning of the experiment, *n* = 2.

Based on the average data, after 24 months soil pH was 11.8% higher than after 3 months. Fertilization with NPK fertilizers resulted in lower pH, which was 13.6% and 16.9% lower in the fertilized groups compared to unfertilized (in S and M systems, respectively). A 5 t/ha biochar dose increased soil pH by 1.72% and 15 t/ha—by 6.84%, compared to the variants without biochar (Figure 6).

Regardless of the tillage-fertilization system, after 3, 6, 12, and 24 months 15 t/ha biochar dose increased soil pH by 12.5%, 5.88%, 5.75%, and 3.98%, respectively. Biochar effect on soil pH was significant in all tillage-fertilization systems (*p* < 0.05) (Table 3). Biochar usage in the M system was more promising than in S. In the M system 15 t/ha biochar dose increased soil pH by 8.79–19.1% (M-1 and M-2) compared to the control group. After 6, 12, and 24 months of biochar incorporation, direct drilling soil had the highest pH (7.31; 7.82; 7.99, respectively).

### 3.3. Soil Electrical Conductivity

**Figure 7:**
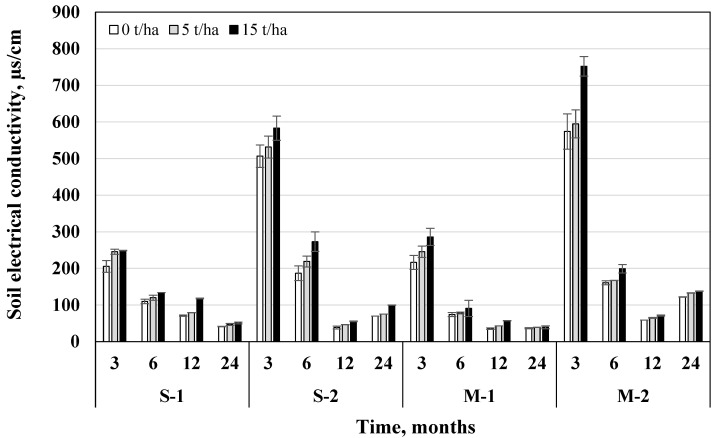
Influence of biochar on the changes of soil electrical conductivity in different tillage-fertilization systems (S—ploughless shallow tillage, M—direct drilling, 1—unfertilized, 2—fertilized) after 3, 6, 12, and 24 months from the beginning of the experiment, *n* = 2.

After 24 months soil electrical conductivity (EC) was on average 82.3% lower than after 3 months. Fertilization with NPK fertilizers resulted in higher soil EC. Fertilization governed 82.1% and 141% higher soil EC compared to unfertilized soil (in S and M systems, respectively). A 5 t/ha biochar dose increased EC by 13% and 15 t/ha—by 23.4%, compared to the variants without biochar (Figure 7).

Regardless of the tillage-fertilization system, after 12 months 5 t/ha and 15 t/ha biochar doses increased soil EC by 9.15–21.6%. The influence of biochar on soil EC was essential in all tillage-fertilization systems (*p* < 0.05) (Table 4). 15 t/ha biochar dose increased soil EC by 28.3–15.8% in the S system (both without fertilization and fertilization, respectively), and in the M system—by 33.9–23.8% compared to variants without biochar. Usage of a 15 t/ha biochar dose in combination with mineral fertilizers determined the highest soil EC in the M system (289 µs/cm). After 3 and 24 months after biochar incorporation, soil variant with direct drilling + fertilization + 15 t/ha biochar dose had the highest EC (752 µs/cm; 137.6 µs/cm).

Thus, the results demonstrate that tillage and fertilization, by directly determining the physical condition of the soil, also determine its electrical conductivity and plant nutrition conditions. Both soil type and land usage have a significant influence on the overall macroporosity, its surface area, and the distribution of pores belonging to the macropore group. Accordingly, the number of macropores, as well as the number of mesopores and their distribution, is an important factor in determining the amount of water in those pores and their electrical conductivity [35,36].

Based on the soil salinity classes, the studied soil groups were characterized as non-saline, as EC values for all groups ranged from 0 to 2 dS/m (from 0.04 dS/m in M-1 after 12 months to 0.75 dS/m in M-2 after 3 months). Soils with an EC less than 2 dS/m are considered non-saline and this does not affect many cereal yields and soil microbiological processes. Even mild to moderate salinity can inhibit grain growth. Salts are a natural component of the soil, but when the concentration of salts in the soil is high, especially close to the roots of plants, the roots attract and absorb less moisture [37]. When the salinity of the soil is high enough, the plant will dry out and die, regardless of the amount of extra water used.

### 3.4. Surface Functional Groups of Soil

**Figure 8:**
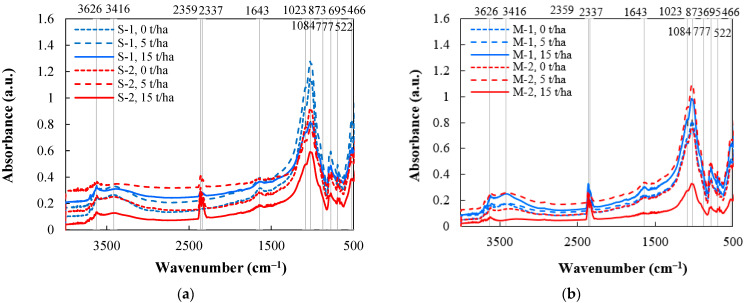
Impact of biochar on the soil surface functional groups in different tillage-fertilization systems after 3 months from biochar application: (**a**) S—ploughless shallow tillage, (**b**) direct drilling, 1—unfertilized, 2—fertilized, *n* = 2.

According to the results of FTIR analysis, after 3 months from biochar application, in both S and M systems, FTIR spectra were similar and had such functional groups: alcoholic –OH (3626 cm^−1^), alkoxy C–O (1023–1084 cm^−1^), aromatic C–H (777–873 cm^−1^) and C=C (1643 cm^−1^) (Figure 8). Comparing fertilized soils amended with different biochar rates, it was observed that in both tillage systems the 5 t/ha biochar rate caused the highest number of functional groups (due to the higher intensity of infrared radiation absorption) and 15 t/ha rate determined the least amount. Meanwhile, in unfertilized soil of the S system, absorption peaks were stronger at 1023–1084 cm^−1^ and 466–522 cm^−1^ wavenumbers under 5 t/ha rate application. In the M system, all absorption peaks were stronger when a 15 t/ha biochar rate was used. We suppose that soil fertilization governs more stable soil structure in different soil tillage systems irrespective of biochar application; meanwhile, the chemical structure of unfertilized soil strongly varied independently of the biochar rate.

The –OH group in soil is related with kaolinite clay minerals (3694, 3620, 3526 cm^−1^), Si–O group with silicates (1031 cm^−1^) and Al–Al–OH with aluminium compounds (913 cm^−1^) [38]. Similarly, in this study, peaks at 3626, 3416, 1023, and 873 cm^−1^ wavenumbers were observed and it shows the existence of O, H, Al, and Si compounds in the soil structure. The peak at 471 cm^−1^ wavenumbers is related to the amount of Si [39]. FTIR spectra of soils showed deposition of aluminosilicates on the incorporated biochar. FTIR spectra demonstrate that all studied soil groups undistinguished of hydrophobic C–H methyl and methylene functional groups (absence of peaks at 2920 cm^−1^ and 2860 cm^−1^, respectively [40]). Meanwhile, C–O functional groups (peaks occur at 1600–1740 cm^−1^ wavenumbers) are related to hydrophilicity. In our experiment, they were found in all soil groups (peaks at 1643 cm^−1^ wavenumber). Soil hydrophilicity increases with an increase in the density of polar functional groups (such as –OH, –COOH, and –NH_2_), but decreases with the increase of density of nonpolar functional groups (–CH_3_ and =CH_2_) [41].

### 3.5. Soil Water Holding Capacity

**Figure 9:**
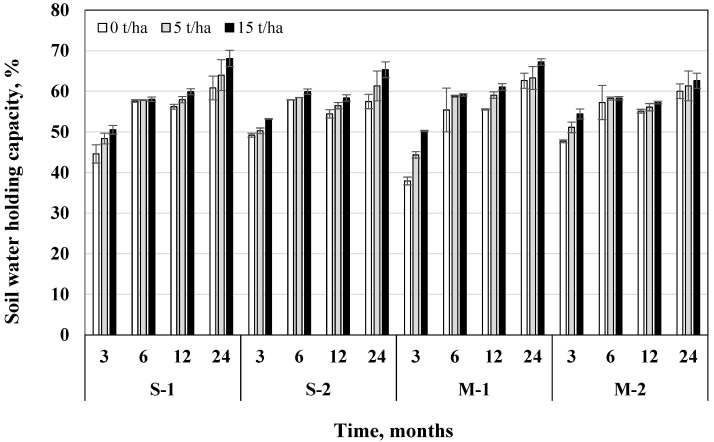
Influence of biochar on changes of soil water holding capacity in different tillage-fertilization systems (S—shallow no-till tillage, M—direct sowing, 1—unfertilized, 2—fertilized) after 3, 6, 12, and 24 months from the beginning of the experiment, *n* = 2.

After 24 months, soil water holding capacity (WHC) was on average 29.6% higher than after 3 months. Fertilization resulted in a slightly higher soil WHC in the M system, which was 0.72% higher than without fertilization. Incorporation of 5 t/ha biochar dose increased WHC by 4.27% and 15 t/ha by 8.48%, compared to the variants without biochar (Figure 9).

Regardless of the tillage-fertilization system, after 3, 6, 12, and 24 months 15 t/ha biochar dose increased WHC significantly more than 5 t/ha (by 16.2%, 3.18%, 6.88%, and 9.25%). Thus, the positive effect of both doses on the increase in soil WHC continued—it remained significantly higher at a 15 t/ha dose. The influence of biochar on WHC was significant in all tillage-fertilization systems (*p* < 0.05) (Table 5). The usage of biochar in M was more promising for increasing WHC. In the S system (both without fertilization and fertilization), 15 t/ha biochar dose increased WHC by 12.4–5.69% in the M system in comparison with variants without biochar. After 3, 12, and 24 months, the variant with direct drilling + 15 t/ha biochar dose had the highest WHC (54.4%, 61.1%, 67.2%). In summary, it can be seen that over time, soil WHC enhancement by biochar gradually increases.

It is known that WHC and water availability to plants in clayey and sandy loams can be improved with biochar addition [42]. A study driven by Yu et al. [43] showed that a high percentage of biochar in soil mixture dramatically increases soil WHC. These results suggest that biochar has the potential to mitigate droughts and increase crop yields in sandy loam [43]. Novak et al. [44] reported an increase in the WHC of sandy loam with 2% of biochar made from grass. It was estimated that when the sandy loam WHC was 16%, yellow pine biochar was able to retain 2.7 times its mass (=270%). A study driven by Yu et al. [43] showed that biochar increased soil WHC by 1.7% of its mass each time 1% biochar was added. The results of these studies are important since biochar is an efficient medium for increasing soil irrigation efficiency, mitigating runoff, and reducing non-point agricultural pollution. The ability of biochar to increase soil WHC is particularly important in drought-prone areas. Some studies have not shown a significant effect of biochar incorporation on moisture retention in sandy loam and coarse-textured sandy soil in field studies. This may have been due to the special hydrophobicity of the biochar, which prevented water from infiltrating the biochar pores. The efficiency of biochar in increasing soil water retention will decrease if biochar is hydrophobic; however, the hydrophobicity of biochar is often removed after environmental exposure.

When assessing the impact of tillage methods on increasing soil WHC, some studies indicate that non-cultivated agriculture is more favorable. An 8-year study driven by Raczkowski et al. [45] evaluating sandy loam showed that no-till farming developed higher bulk density, lower total porosity, and macroporosity, but higher capillary porosity (microporosity) and WHC than conventional farming.

### 3.6. Soil Moisture Content

**Figure 10:**
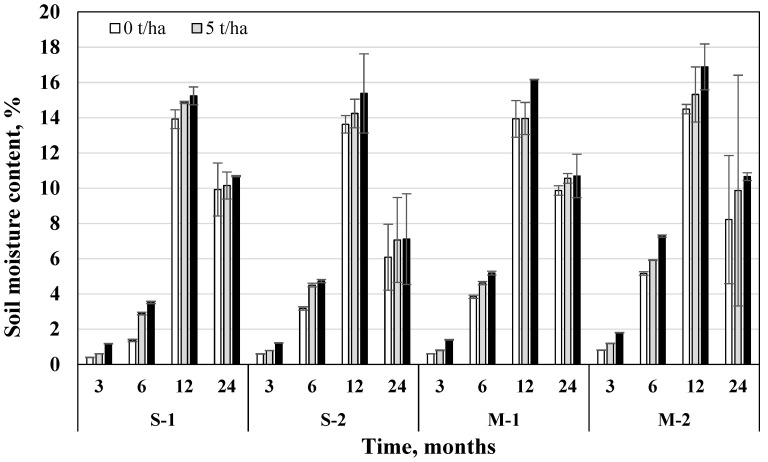
Influence of biochar on changes in soil moisture content in different tillage-fertilization systems (S—shallow no-till, M—direct sowing, 1—unfertilized, 2—fertilized) after 3, 6, 12, and 24 months from the beginning of the experiment, *n* = 2.

Based on average data, after 24 months, soil moisture content (MC) was 872% higher than after 3 months. Fertilization resulted in 6.52% higher MC in the M system than without fertilization. In the shallow ploughless tillage system, MC was on average 7.36% lower in fertilized soil compared without fertilization. The incorporation of 5 t/ha biochar increased MC by 10.7% and 15 t/ha—by 21.8%, compared to the variants without biochar (Figure 10).

Regardless of the tillage-fertilization system, after 3, 6, 12, and 24 months, 15 t/ha biochar dose increased MC significantly more compared to 5 t/ha dose (by 133%; 52.9%; 13.8%; 14.8%). The influence of biochar on MC was significant in all tillage-fertilization systems (*p* < 0.05), except for the combined effect of different factors (Table 6). The usage of biochar in the M system was more promising for increasing soil MC than S. If in the shallow ploughless tillage + fertilization system 15 t/ha biochar dose increased MC by 21.2%, then in the direct drilling system—by 27.7% compared to variants without biochar. After 3, 6 and 12 months the highest soil MC was obtained with direct drilling + fertilization + 15 t/ha biochar dose (1.8%; 7.28%; 16.9%). Soil moisture retention is generally higher in a no-tillage system than in conventional tillage. Non-arable agriculture has the advantage of preserving the soil from wind erosion and promoting the retention of soil moisture.

### 3.7. Soil Wettability

Based on soil wettability results, it can be seen that all the studied soil groups in M and S systems showed high wettability (WDPT ≤ 1 s) or slight hydrophobicity (WDPT = 2) after 6 months and after 12 months (Table 7). According to Chenu et al. [5], soils with instantaneous wettability (WDPT ≤ 1 s) are considered hydrophilic. It can be stated that the influence of biochar, fertilization, and tillage methods on soil wettability is stable for 6 months period. Other studies have estimated that, over time, soil organic matter fills the pores of biochar and reduces its specific surface area. Ren et al. [46] found that after 0.5 years the surface area of biochar in agricultural soil increased and after 1.5 years decreased. Biochar, which has a higher specific surface area, has better sorption for water.

A study driven by Ojeda et al. [47] assessed that after 1 year, the soil-biochar mixture was considered hydrophilic because the contact degree values were less than 90°. There were no differences between collection times and this suggests that the impact of biochar on soil wettability is stable over a 1-year period. When comparing the control soil with the biochar mixtures, wettability was not significantly affected by the biochar dose.

### 3.8. Triticale Grain Yield and Correlation Analysis

**Figure 11:**
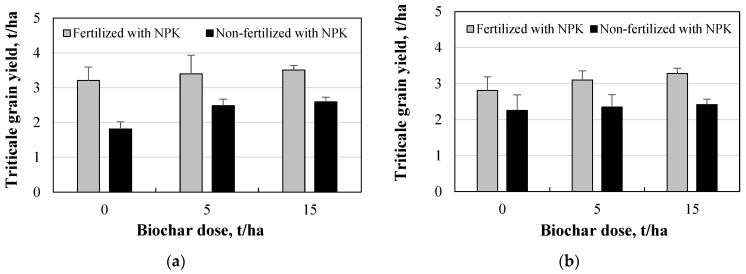
Triticale grain yield of standard moisture: (**a**) M—direct drilling; (**b**) S—shallow ploughless tillage.

The results showed a significant benefit of soil fertilization with NPK mineral fertilizers for triticale grain yield in both tillage systems (Figure 11). Significant differences between fertilized and non-fertilized soil groups were found when evaluating both tillage methods (*p* < 0.05). The highest yield of standard moisture triticale grain (3.51 t/ha) was determined in the system of direct drilling, fertilization, and 15 t/ha biochar dose (Figure 11a). This result may have been due to better nutritional conditions of the plants in the fertilized soil, which was determined by the usage of liquid fertilizers. Macro- (N, P, K, Ca) and microelements (Cu, Mn, Zn, B, Fe, Mo) also play an important role in plant growth and development, which in turn increases plant growth and yield. The incorporation of biochar resulted in an increase in triticale yield in all tillage-fertilization systems, the largest of which was in the case of direct drilling and non-fertilization at 5 and 15 t/ha biochar rates (36.8% and 42.8%, respectively).

A study driven by Terzic et al. [48] similarly found that the mean triticale yield was lowest in the unfertilized control group (2.06 t/ha) and significantly higher in the fertilized groups (4.05 t/ha, NP2K effect; 4.11 t/ha, NP1K effect). In the mentioned study, the highest grain yield was obtained in the NP1K variant (120 kg/ha nitrogen fertilizer content; 60 kg/ha phosphorus (P_2_O_5_) fertilizer content, and 60 kg/ha potassium (K_2_O) fertilizer content). Study driven by Gebremedhin et al. [49] showed that the incorporation of biochar into the soil increases the yield of wheat grain and straw by 15.7% and 16.5%, respectively. A study driven by Bielski et al. [19] showed that the control group (without nitrogen fertilizers), which was 3.17 t/ha, had the lowest triticale yield. The highest yield was observed in the effect group of the highest amount of nitrogen fertilizers (160 kg/ha) in the first study year (2013), which amounted to 5.17 t/ha. The grain yield of winter triticale strongly depended on the weather conditions during the whole study year and on the amount of nitrogen fertilizers. Previous authors pointed out that the triticale yield depended not only on fertilization but also on weather conditions. Some researchers point out that air is one of the most important factors influencing grain yields. A study driven by Gibson et al. [50] showed that the application of nitrogen fertilizers (33 kg/ha) increased the yield of triticale grain by 64% compared to the control group and reached 3.7 mg/ha after 2 years.

**Figure 12:**
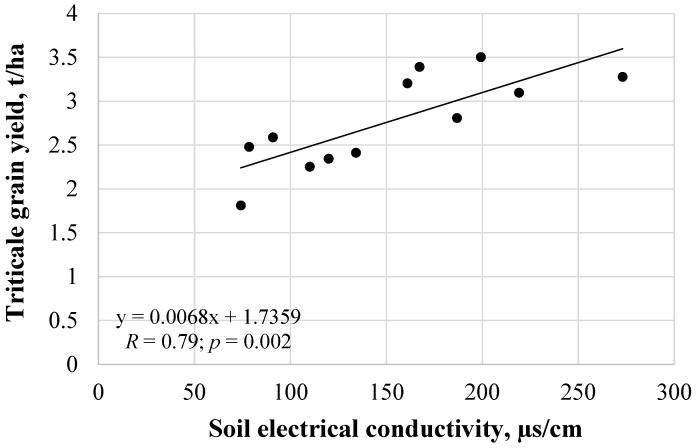
Relationship between soil electrical conductivity (µs/cm) and triticale grain yield (t/ha).

Pearson correlation analysis showed a significant relationship between triticale grain yield and its soil electrical conductivity (*R* = 0.79; *R*^2^ = 0.62; *p* = 0.002) (Figure 12). As the electrical conductivity of the soil increased, the yield of triticale also increased. Soil electrical conductivity can provide guidelines for assessing soil productivity. Therefore, in the future, it is necessary to clarify the mechanisms between the transfer of macro- and micronutrients to agricultural crops from soils improved with mineral fertilizers in combination with biochar. 

Plant yields strongly depend on the soil conditions in which the plant root system develops. The quality of soil conditions is defined as the appropriate air-water regime, mechanical composition, and soil nutrient resources [51]. A study driven by Zhao et al. [52] showed that there was a linear correlation between soil electrical conductivity and winter wheat yield at different wheat growth stages. The coefficients of determination of the models were all greater than 0.63. The strength and direction of the relationship between cereal yield and soil electrical conductivity depend on the amount of precipitation encountered in the early growing season. Cereal yields correlated strongly and negatively with electrical conductivity when there was low precipitation in March. Meanwhile, yields were positively or weakly negatively correlated with electrical conductivity when there was low to moderate precipitation in March [53]. Similarly, in this study, in early cultivation, in March, precipitation was on average lower (12.6 mm) than in July (22 mm) or August (35.7 mm), and lower precipitation in spring can be attributed to a strong positive soil electrical conductivity and correlation of triticale yield. Higher nutritional values of soil may be due to higher nutrient levels, so a positive relationship between cereal yield and soil electrical conductivity can also be attributed to potentially higher nutrient levels. This is because the soil can conduct an electric current due to the movement of ions in solution, and these mobile ions are determined by the availability of nutrients as a function of crop yield. Thus, the electrical conductivity of the soil is expected to be higher where the concentration of nutrients required for crop growth and yield is higher.

## 4. Discussion and Future Research

Previous studies found that more hydrophobic soils have a higher amount of organic matter. According to the study of Mirbabaei et al. [54], SOM, soil texture, and pH revealed significant relation with its hydrophobicity. The positive correlation (*r* = 0.42) between logWDPT and the amount of SOM was found in all tested samples. Other studies found a positive correlation between the amount of organic matter and hydrophobicity in clay soil from the Utah State (USA) [55], in pine forest soil affected by wildfires from Spain [56] and in Mexican volcanic soils as well [57]. Though Vogelmann et al. [6] determined very small coefficients between SOM and hydrophobicity; they concluded that soils were more hydrophobic having a higher amount of organic matter. In this study, irrespective of SOM content in sandy loam soil and tillage-fertilization practice, the soil was hydrophilic during the whole period of investigations. It can be explained by their similar chemical composition in which oxidized aluminum and silica components and hydrophilic C–O groups dominated, according to the molecular spectrometric method. 

The soil moisture content (MC) is usually determined by the soil texture and precipitation. Previous studies found a positive impact of biochar on the soil MC and increase of water holding capacity (WHC) which was better expressed in sandy soils. It was shown that soils having a higher fraction of clay particles gained less benefit from the incorporation of biochar compared to sandy soils [58]. The meta-analysis of 103 studies showed a higher biochar effect on sandy soils than clay soils and on acidic soils than on neutral soils [59]. A positive impact of biochar on the increase in the soil WHC was determined in Amazon *Anthrosol* which was 18% higher compared to the soil without biochar [60]. It confirms the idea that demand for soils irrigation could potentially decrease after the application of biochar. It was also determined that biochar having a sufficient amount of humic compounds can increase soil WHC [61]. The potential of biochar to retain moisture in the soil is significant and it is very important in agricultural regions where climate change will further impact its water resources. Assessing the soil tillage impact on the soil WHC, it had a tendency to increase in zero-tillage (direct drilling into untilled and/or into minimum tilled soil) and at the same time, it increased available water content for plants [62]. A study driven by Fernandez-Ugalde et al. [63] found that the water retention of soil was up to 23% higher in zero-tillage compared to conventional tillage. Similarly, in this study, direct drilling revealed better results of the WHC—it was 11% higher compared to ploughless shallow tillage. 

Inherently, biochar is a very porous material and, when its initial hydrophobicity is lost, it potentially oxidizes, absorbs, and retains water [58]. A study driven by Yi et al. [64] which used low-temperature (400 °C) hydrophobic poultry excrement biochar and hydrophilic agricultural sandy soil mixture found that even small amounts of biochar amendment into the soil increased its hydrophobicity (variation of contact angles from 8° in the control soil and until 20° after 2% of biochar addition). However, when biochar was additionally washed with deionized water and heated at the temperature of 105 °C for 24 h, it rendered from hydrophobic to hydrophilic. Therefore, the hydrophilicity of hydrophobic pine wood biochar and hydrophilic soil mixture can be explained by the impact of natural abiotic factors (higher precipitation and higher average air temperature) in this study. Hajnos et al. [65] studied how the wettability of soils differed according to the water drop penetration time (WDPT) test and found that peat soil (*Haplosol*) had higher WDPT than 1 h, which shows their extreme hydrophobicity, meanwhile, mineral soil had WDPT less than 5 s and then classified as hydrophilic [65]. Obtained results confirmed the statements that constantly soils with permanent plant cover have lower wettability than arable agricultural soils. Hydrophobicity of soils is increased by the plants’ excreted oils and waxes which are hardly decomposed. The wettability of soil also heavily depends on its mineral composition. Lourenco et al. [66], using the method of contact angle analysis, found that sulphides among 21 soil minerals were characterized as hydrophobic (>99°), meanwhile silicates showed a hydrophilic tendency which varied from 0° (quartz) until 54° (calcite). In this experiment, the FTIR analysis showed that silicates were the dominant soil mineral group: it showed the strongest peaks at 1023 cm^−1^ wavenumber which indicate the presence of Si–O compounds. Exactly silica tetrahedron (SiO_4_)^4−^ is a fundamental unit of all silicates.

Long-term experiments, evaluating the impact of different biochar types, application rates, tillage-fertilization systems, and soil types on the agronomical efficiency can be the ideal way to test whether the interaction between soil structure and water can be permanently affected by the addition of biochar. Grassy biomass feedstock compared to widely available woody feedstocks can offer more positive results on the available water content for plants [67]. Hemp can be one of such feedstocks and, according to this study, the low-temperature (450 °C) hemp biochar had a higher specific surface area (BET = 39.4 m^2^/g) and wettability (WDPT = 2.67 s) compared to biochar types made from other feedstocks. It is likely that interactions between biochar-soil develop with time when biochar further oxidizes and contributes to the formation and stabilization of soil aggregates. Soil aggregates are an important part since they govern the stability of organic matter; improve the structure of their pores and the solid part [68]. The selection of biochar types and rates is an important agricultural measure for sustainable soil usage.

## 5. Conclusions

The combined effect of 15 t/ha biochar dose and NPK fertilizers in a direct drilling system improved soil’s physical-chemical properties and the triticale yield (3.51 t/ha) the most. This can be explained by better nutritional conditions for plants from fertilized soil due to the incorporation of macroelements (N, P, K) with fertilizers and microelements (Zn, Cu) with biochar addition, which increases plant growth and yield. A significant relationship was found between triticale grain yield and soil electrical conductivity when the correlation coefficient was 0.79. This indicates that with higher soil electrical conductivity, the soil contains more nutrients leading to better triticale growth and grain yields. Based on average data from the whole 24-month experiment, 15 t/ha biochar dose increased soil organic matter content by 33.7%, pH—6.84%, electrical conductivity—23.4%, water retention capacity by 8.48%, and moisture content by 21.8% compared to the variants without biochar. Irrespective of hydrophobic biochar dose, the analyzed sandy loam remained hydrophilic or slightly hydrophobic after 24 months due to low water drop penetration times (1–2 s). Its hydrophilicity can be attributed to hydrophilic hydroxide (–OH) and silicon oxide (Si–O) functional groups, which were found using infrared spectrum at 3626, 3416, 1023, and 466 cm^−1^ wavenumber.

## Figures and Tables

**Table 1:** Physical-chemical properties of low-temperature pine wood biochar, *n* = 3, ±standard deviation.

Initial Feedstock	Pyrolysis Temperature (°C)	Holding Time (h)	pH_KCl_	Water Holding Capacity (%)	H (%)	N (%)	Cation Exchange Capacity (cmol_c_/kg)
Pine wood	450	2	8.53 ± 0.13	449 ± 8.08	11.07 ± 0.19	0.25 ± 0.006	0.68 ± 0.11

H—hydrogen; N—nitrogen.

**Table 2:** Variance analysis of soil organic matter content, *n* = 3.

Factors and Their Interactions	F-Fact	Least Significant Difference LSD_05_	Probability P
Factor A—soil tillage-fertilization system	363.23 **	0.044	0.000000
Factor B—biochar dose	880.85 **	0.036	0.000000
Factor C—date of investigation	1319.57 **	0.044	0.000000
A×C	334.3 **	0.083	0.000000
B×C	235.48 **	0.076	0.000000
A×B	76.59 **	0.076	0.000000
A×B×C	52.6 **	0.125	0.000000

**—*p* < 0.01.

**Table 3:** Variance analysis of soil pH, *n* = 3.

Factors and Their Interactions	F-Fact	Least Significant Difference LSD_0.5_	Probability P
Factor A—soil tillage-fertilization system	2383.28 **	0.024	0.000000
Factor B—biochar dose	361.74 **	0.02	0.000000
Factor C—investigation date	789.9 **	0.024	0.000000
A×C	31.07 **	0.046	0.000000
B×C	22.69 **	0.042	0.000000
A×B	135.28 **	0.042	0.000000
A×B×C	8.87 **	0.069	0.000000

**—*p* < 0.01.

**Table 4:** Variance analysis of soil electrical conductivity, *n* = 3.

Factors and Their Interactions	F-Fact	Least Significant Difference LSD_0.5_	Probability P
Factor A—soil tillage-fertilization system	345.21 **	6.82	0.000000
Factor B—biochar dose	29.46 **	5.57	0.000000
Factor C—investigation date	1755.79 *	6.82	0.000000
A×C	145.69 **	12.9	0.000000
B×C	3.54 **	11.8	0.000000
A×B	4.72 **	11.8	0.000000
A×B×C	6.61 **	19.3	0.000000

**—*p* < 0.01; *—*p* < 0.05.

**Table 5:** Variance analysis of soil water holding capacity, *n* = 3.

Factors and Their Interactions	F-Fact	Least Significant Difference LSD_0.5_	Probability P
Factor A—soil tillage-fertilization system	3.3 **	0.324	0.000000
Factor B—biochar dose	199.26 **	0.265	0.000000
Factor C—investigation date	1004.82 *	0.324	0.000000
A×B	34.4 **	0.612	0.000000
B×C	13.27 **	0.562	0.000000
A×B	4.89 **	0.562	0.000000
A×B×C	3.28 **	0.917	0.000000

**—*p* < 0.01; *—*p* < 0.05.

**Table 6:** Variance analysis of soil moisture content, *n* = 3.

Factors and Their Interactions	F-Fact	Least Significant Difference LSD_0.5_	Probability P
Factor A—soil tillage-fertilization system	18.91 **	0.273	0.000000
Factor B—biochar dose	27.68 **	0.223	0.000000
Factor C—investigation date	1452.77 *	0.273	0.000000
A×C	11.19 **	0.514	0.000000
B×C	1.26	0.472	0.282342
A×B	0.57	0.472	0.754204
A×B×C	0.3	0.771	0.997114

**—*p* < 0.01; *—*p* < 0.05.

**Table 7:** Soil wettability assessed by water drop penetration time test under ploughless shallow tillage unfertilized (S-1) and fertilized (S-2), direct drilling unfertilized (M-1) and fertilized (M-2) soil, *n* = 3.

Soil Tillage-Fertilization System	M-1			M-2			S-1			S-2		
Biochar Dose, Research Date	0 t/ha	5 t/ha	15 t/ha	0 t/ha	5 t/ha	15 t/ha	0 t/ha	5 t/ha	15 t/ha	0 t/ha	5 t/ha	15 t/ha
3 months	≤1	≤1	≤1	≤1	≤1	≤1	≤1	≤1	≤1	≤1	≤1	≤1
6 months	≤1	≤1	≤1	≤1	≤1	≤1	≤1	≤1	≤1	≤1	≤1	≤1
12 months	2	2	2	1	1	1	2	2	2	1	1	1
24 months	1	1	1	1	1	1	1	1	1	1	1	1

S—ploughless shallow tillage; M—direct drilling.

## Data Availability

All data included in the main text.
